# Multiple or More Severe Grade Prevalent Vertebral Fractures Are Associated with Higher All-Cause Mortality in Men with Nonmetastatic Prostate Cancer Receiving Androgen Deprivation Therapy

**DOI:** 10.3390/cancers17132131

**Published:** 2025-06-25

**Authors:** Kashia Goto, Daisuke Watanabe, Hiromitsu Takano, Kazuki Yanagida, Norikazu Kawae, Hajime Kajihara, Akio Mizushima

**Affiliations:** 1 Department of Palliative Medicine, Juntendo University Graduate School of Medicine, Tokyo 113-8421, Japan; k.goto.ew@juntendo.ac.jp (K.G.); n.kawae.sh@juntendo.ac.jp (N.K.); akiom@juntendo.ac.jp (A.M.); 2 Department of Urology, Koto Hospital, Tokyo 136-0072, Japan; k.yanagida.sr@juntendo.ac.jp; 3 Department of Molecular and Cellular Therapeutics, Juntendo University Graduate School of Medicine, Tokyo 113-8421, Japan; 4 Department of Orthopedic Surgery, Koto Hospital, Tokyo 136-0072, Japan; hrtakano@juntendo.ac.jp (H.T.); h.kajihara.jk@juntendo.ac.jp (H.K.); 5 Department of Orthopedic Surgery, Juntendo University Graduate School of Medicine, Tokyo 113-8421, Japan

**Keywords:** cancer treatment-induced bone loss (CTIBL), prevalent vertebral fractures (PVFs), Semiquantitative (SQ) method, androgen deprivation therapy (ADT), nonmetastatic prostate cancer (nmPC)

## Abstract

**Background/Objectives**: Prognostic information for nonmetastatic prostate cancer (nmPC) patients with prevalent vertebral fractures (PVFs) is very limited. Vertebral fractures can impair physical function, limit activities of daily living, and decrease quality of life. Prevention of vertebral fractures may be important to improve patient prognosis. This study aims to investigate the impact of the presence and severity of PVFs on overall survival in patients with nmPC undergoing androgen deprivation therapy (ADT). **Methods**: A total of 275 men (median age: 73 years) with nmPC who underwent ADT were studied retrospectively. The median observation period was 55 months. Variables included age, body mass index, T classification, N classification, Gleason score, and pretreatment serum prostate-specific antigen levels. PVF was diagnosed from the sagittal computed tomography images of Th1 to L5 before initiating ADT, and the severity was determined by the number of PVFs and the Semiquantitative (SQ) method. Hazard ratios and 95% confidence intervals for overall survival were calculated using the Cox proportional hazards model. **Results**: During the observation period, 30 patients died from all causes. Multivariate Cox regression analysis identified multiple PVFs and high-grade PVFs, as determined by the SQ method, as significant predictors of overall survival. The analysis utilized two adjustment models: one adjusted for age only and the other adjusted for age, Gleason score, and clinical T stage. **Conclusions**: Multiple PVFs and high-grade PVF determined by the SQ method prior to ADT initiation were associated with higher all-cause mortality in nmPC patients treated with ADT.

## Simple Summary

Androgen deprivation therapy (ADT) is an established effective treatment for patients with nonmetastatic prostate cancer. However, cancer treatment-induced bone loss (CTIBL) and the associated increase in fractures are recognized as significant complications that affect patient prognosis. Current guidelines for CTIBL management recommend a bone health assessment prior to initiating ADT, which includes the identification of prevalent vertebral fractures (PVFs). While fractures occurring after ADT have been reported to be associated with reduced overall survival, the relationship between PVFs before initiating ADT and actual prognosis remains unclear. This study investigates the impact of the presence and severity of PVFs on overall survival in patients with nonmetastatic prostate cancer undergoing ADT. Additionally, it provides valuable insights into the clinical usefulness of spine health assessment before initiating ADT.

## 1. Introduction

Androgen deprivation therapy (ADT), achieved through surgical or drug castration, is widely used because prostate cancer exhibits androgen-dependent growth. ADT has become a key effective treatment for nonmetastatic prostate cancer (nmPC) patients. However, the decrease in bone density and quality, as well as the increase in fractures, are known adverse events associated with the treatment [1,2]. Cancer treatment-induced bone loss (CTIBL) leads to bone fractures and is associated with a decrease in the quality of life of cancer survivors. Additionally, fractures occurring after ADT have been reported to be associated with shorter overall survival (OS) [3]. Therefore, current national and international guidelines recommend bone health assessment prior to the initiation of ADT to prevent fractures due to CTIBL.

Current guidelines on CTIBL management recommend bone health assessment prior to the initiation of ADT, including confirmation of prevalent vertebral fractures (PVFs). Regarding changes in daily functional abilities due to vertebral compression fractures, it is believed that spinal deformities and lower back pain affect physicla function, activities of daily living (ADL), and social participation. However, there are few studies that have validated this. Nonetheless, several studies have suggested an association between vertebral fracture severity and daily activity. Population-based surveys indicate that undiagnosed vertebral fractures are correlated with diminished physical and functional status, reduced capacity to perform ADL, and overall decreased patient well-being [4,5,6,7,8,9].

In a study conducted by GG Crans et al. utilizing the Semiquantitative (SQ) method to assess the impact of vertebral fractures on quality of life, it was found that vertebral fractures classified as SQ grade 3 (SQ 3) were significantly associated with lower overall health-related quality of life (HRQOL) scores [10]. Additionally, these fractures indicated significantly lower physical function, symptoms, and emotional scores. On the other hand, there is very limited information on the prognosis of nmPC patients with PVFs compared to studies in postmenopausal women. Wang Y et al. noted that high daily physical activity increases cancer-specific survival rates in patients diagnosed with prostate cancer [11]. A study by Ofelein et al. demonstrated that prostate cancer patients who experienced fractures during treatment had poor prognosis compared to those without fractures. Additionally, skeletal fractures in these patients were found to be an independent and adverse predictor of survival [3]. Although these studies acknowledge reports that fractures occurring after ADT are associated with shorter overall survival, the association between PVF before the initiation of ADT and actual prognosis has not yet been clarified.

Therefore, we designed this study to evaluate the impact of the presence and severity of PVF using the SQ method assessed by computed tomography (CT) before the initiation of ADT on the survival rate in men with nmPC.

## 2. Materials and Methods

### 2.1. Study Population

This study was designed as a retrospective single-center cohort study. A total of 275 men diagnosed with prostate cancer and treated with ADT at Koto Hospital (Koto-Ku, Tokyo, Japan) between January 2018 and February 2022 were evaluated retrospectively. Clinicopathologic factors such as age, body mass index (BMI), Tumor Node Metastasis (TNM) classification, Gleason score, and prostate-specific antigen (PSA) levels were investigated. CT and bone scintigraphy were used to confirm nonmetastatic prostate cancer. The absence of a mass lesion in the bone could be confirmed by CT scan. During the period of this study, all patients diagnosed with osteoporosis based on their latest dual-energy X-ray absorptiometry (DXA) scan (GE Lunar Corp., Madison, WI, USA) received bone mineral density (BMD) interventions. These treatments were administered either by the attending physician or through consultations with an orthopedic surgeon. All protocols of this study were approved by the review board and ethics committee of Koto Hospital (No. 202221) and conformed to the tenets of the Declaration of Helsinki.

### 2.2. Bone Assessment and Blood Sampling

For image evaluation, a spinal surgeon reviewed sagittal CT images from the chest to the pelvis taken before the initiation of ADT. Using sagittal CT images from Th1 to L5, PVFs prior to ADT initiation were diagnosed, and the number and severity of fractures were assessed using the SQ method.

SQ assessment involves classifying vertebral fractures from Grade 0 to 3. Vertebral fractures are identified when they fall into Grade 1 or higher. The grading is as follows: Grade 0 indicates normal, non-fractured vertebrae; Grade 1 indicates mild fractures; Grade 2 indicates moderate fractures; and Grade 3 indicates severe fractures.

The anterior, central, and posterior heights of each vertebral body from Th1 to L5 were measured, and if at least one of these three measurements was reduced by 20% or more compared to the height of the nearest non-compressed vertebral body, it was diagnosed as a vertebral fracture. The severity of PVF was classified as Grade 0 to 2 or higher [12]. Additionally, clinical data, including age, body height, weight, BMI, serum creatinine levels, eGFR, TRACP-5b, PSA, and medical history (Diabetes mellitus, Hypertension, Dyslipidemia) were collected.

### 2.3. Statistical Analysis

Using the Cox proportional hazards model, we examined the impact of clinicopathological factors and the severity of PVFs on all-cause mortality. The impact on prostate cancer-specific mortality was not examined due to the low number of outcomes (death due to prostate cancer: 6 out of 275 patients), which compromised the reliability of the analysis. The rate of all-cause mortality was calculated using the Kaplan–Meier method, and comparisons based on the severity of the PVFs were made using the log-rank test. *p* values of <0.05 were considered to indicate statistical significance. All statistical analyses were performed using JMP^®^ 14 (SAS Institute Inc., Cary, NC, USA).

## 3. Results

The patient backgrounds and clinical laboratory values of 275 men diagnosed with nmPC and initiated on ADT at Koto Hospital between January 2018 and February 2022 are shown in Table 1. The median age at prostate needle biopsy was 73 years (range 55–89), the median BMI was 23.3, and the median PSA was 12.4 ng/mL. Data from follow-up imaging and clinical records were available to assess the occurrence of new fractures. No incident vertebral fractures were identified during the follow-up period. Therefore, our analysis focused exclusively on prevalent vertebral fractures identified prior to ADT initiation. The clinical T category distribution was as follows: T1c in 41 patients (14.9%), T2a in 64 patients (23.3%), T2b in 68 patients (24.7%), T2c in 64 patients (23.3%), T3a in 22 patients (8.0%), T3b in 7 patients (2.5%), and T4 in 9 patients (3.3%). The clinical N category was N0 in 251 patients (91.3%). Of the patients, 168 patents (61.1%) had a biopsy Gleason score of 7, constituting the majority. During the study period, 36 patients died, of whom 6 deaths were attributed to prostate cancer and 30 to other causes. Importantly, no cases of clinical metastatic disease were documented throughout the observation period, including among patients with multiple or higher-grade prevalent vertebral fractures. However, due to the retrospective nature of the study, detailed cause-of-death information for non-prostate cancer deaths was not available, and we could not determine whether any of these were indirectly related to undiagnosed metastases.

Overall, 41.8% were treated with ADT alone, 13.8% with ADT/radical prostatectomy (RP), and 44.4% with ADT/radiotherapy (RT). The median duration of observation was 55 months (range 1–150). Most patients underwent ADT for 12 months or less. Those who preferred not to undergo radiation therapy or radical prostatectomy chose to continue ADT throughout the entire treatment period. Patients who were receiving denosumab or bisphosphonates, which can affect fracture risk, were not included in this study. Also, no patient received treatment for PVF during ADT treatment. CT imaging analysis revealed that 54 of 275 patients in this cohort had one or more PVF at baseline: 31 (11.3%) had one fracture, 23 (8.3%) had two or more, and 40 (14.5%) had an SQ grade 1. The number of patients with SQ grade 2 or higher was 14 (5.1%) (Table 1).

**Figure 1:**
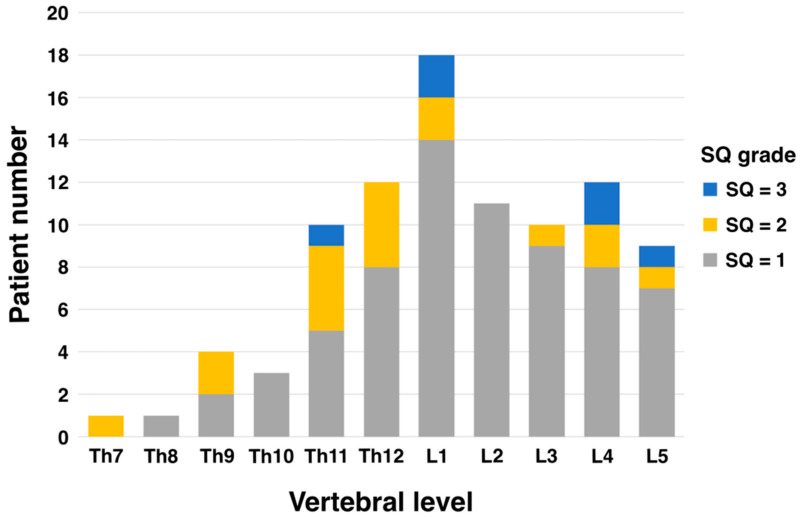
Patient number of vertebral fracture level and SQ grade.

The most common level of vertebral fracture was the first lumbar spine (L1) in 18 patients, 14 of whom had SQ grade 1. The next most common were the fourth lumbar (L4) and twelfth thoracic vertebrae (Th12) with 12 patients each, of which 8 were SQ grade 1 each (Figure 1).

Using a Cox proportional hazards model, the impact of each factor on OS for men with nmPC is presented in Table 2. In univariate analysis, OS tended to be shorter with older age (hazard ratio [HR], 1.1; 95% confidence interval [CI], 1.04–1.17; *p* = 0.0018; Table 2), concomitant PVF, higher number of PVFs, higher SQ grade of PVFs, Gleason score, and clinical T stage (*p* = 0.0003, *p* = 0.0007, *p* = 0.0006, *p* = 0.0225, *p* = 0.0459, respectively; Table 2).

In multivariate analysis, adjusted for age (Model 1) and for age, Gleason score, and clinical T stage (Model 2), two or more PVFs and a high SQ grade of 1 or higher were still significant predictors of OS (Table 3). In Model 2, the statistical correlation between a single PVF and OS weakened.

## 4. Discussion

To prevent fractures associated with CTIBL, the bone health assessment prior to initiating ADT treatment includes an evaluation of the presence or absence of PVFs. The position statement of the ‘Clinical Practice Manual for Cancer Treatment-Induced Bone Loss (CTIBL)’ highlights the rationale for creating this manual [13]. If a patient has a pre-existing vertebral fracture when starting androgen deprivation therapy (ADT), the management guidelines recommend treating the patient for osteoporosis. This includes evaluating bone mineral density (BMD) and other fracture risk factors, diagnosing osteoporosis, and considering effective pharmacological treatments such as denosumab or bisphosphonates. Regular monitoring of BMD, especially for patients with lumbar spine or proximal femoral BMD T scores ≥ −1.5, is also advised. Additionally, patients should be encouraged to maintain adequate calcium and vitamin D intake and engage in moderate exercise to support bone health. This structured approach helps mitigate further fracture risk and ensures comprehensive management of bone health during ADT. It also states that although patients with CTIBL have a 2–3 times higher risk of fractures, the Japanese guidelines for the prevention and treatment of osteoporosis have overlooked these patients, resulting in having missed opportunities for treatment. However, it is also noted that evidence regarding the incidence of fractures, treatment efficacy, and outcomes for CTIBL patients in Japan is insufficient.

There is very limited information on the prognosis of nmPC patients with PVF. In this study, we conducted a retrospective cohort study involving 275 patients with nmPC to evaluate the impact of vertebral fracture assessment prior to initiating ADT on OS after ADT. This study includes nmPC, for which, in most cases, the backbone of the treatment is prostatectomy/radiation therapy with the addition of short/long-term ADT when indicated. However, more than 40% of patients received ADT alone. There are two main reasons why more than 40% of our patients were treated with ADT alone. One is due to the economics of the patient’s decision to undergo treatment, and the other is due to the patient’s own will/preference. The percentage of patients with PVF grade 1 or higher before the initiation of ADT was 19.6%. Although there was no control group, multivariate Cox regression analysis showed that the number of PVFs and SQ grade before the initiation of ADT were significantly correlated with OS after ADT, while age and cancer progression/metastasis were not associated. We believe that our results may provide evidence to support the clinical utility of vertebral fracture assessment prior to the initiation of ADT.

Interestingly, the factor of age and cancer progression/metastasis did not show a significant association with OS after ADT treatment. This may be because 251 patients (91.3%) in this study were classified as clinical N category 0. Additionally, in the T category, there were 22 patients (8%) with T3a, 7 patients (2.5%) with T3b, and 9 patients (3.3%) with T4, indicating that a majority of the patients were in relatively early stages of cancer progression/metastasis. In a study by Shunfa Huang et al., involving 100 prostate cancer patients and a control group of healthy individuals, the SQ method was used to investigate osteoporotic vertebral fractures and their associated factors before and after treatment [14]. The incidence of spinal fractures in prostate cancer patients was 16% before treatment and 31% after treatment, while the incidence in the control group was 29%. The spinal fracture rate in prostate cancer patients before treatment was significantly different from that in the control group and from the rate in prostate cancer patients after treatment. Multivariate logistic regression analysis indicated that age was the primary factor influencing the rate of spine fracture. In their study, the average age of patients was 69.68 years, and the pre-treatment incidence of PVF was 16%. In contrast, our study had a median age of 73 years and a pre-treatment incidence of 19.6%, suggesting that our patient backgrounds were approximately consistent with previous studies. Our results indicate that OS of nmPC patients after ADT is significantly affected not by age but by multiple PVFs and high SQ grade before the initiation of ADT. These findings are consistent with current guidelines that advocate for the clinical utility of bone health assessment (checking for existing fractures) before the initiation of ADT.

Marsha M. van Oostwaard et al. suggested that the prevalence of vertebral fractures in prostate cancer patients may be higher than in the general population [15]. Prostate cancer is characterized by the production and secretion of PSA, which is not produced by non-prostate tissues or carcinomas. Prostate cancer-specific osteoblastic changes are hypothesized to be partially driven by PSA, resulting in a significant shift in bone metabolism towards bone formation. Yonou H et al. reported that PSA is a type of proteolytic enzyme classified as a serine protease [16]. Studies conducted both in vitro and in vivo have shown that PSA itself stimulates the proliferation of osteoblasts and reduces the number of osteoclasts. Bone remodeling is regulated by precise crosstalk between osteoblasts and osteoclasts [17,18]. Although PSA-induced osteoblast predominance may result in apparently high bone density, the imbalance caused by reduced osteoclasts leads to abnormal bone remodeling, which may result in bone fragility. The resulting bone fragility, when considering bone quality and strength, may be reflected in the prevalence of PVFs.

In a study evaluating whether the Vertebral Bone Quality (VBQ) score could predict fragility fractures in a population with low bone density and high fracture risk, it was found that the VBQ score was an independent predictor of fragility fractures in at-risk patients [19]. Moreover, the VBQ score was a better predictor of fracture risk than BMD measured by DXA. This indicates that even in populations with low bone density and high fracture risk, bone quality is a superior predictor of subsequent fracture risk compared to bone density.

On the other hand, the results of a study by Hussain SA et al. in advanced prostate cancer suggested a higher incidence of osteoporosis and osteopenia in men with advanced prostate cancer compared to age-matched controls, even before the initiation of ADT [20]. A large population-based prospective study in Sweden demonstrated that elderly men with elevated high-sensitivity C-reactive protein (hs-CRP) were at increased risk of fractures and that these fractures were primarily vertebral fractures [21]. In this study, the association between hs-CRP and fractures was independent of BMD. Hussain SA et al. included patients with advanced prostate cancer [20]. Even though patients with advanced prostate cancer have a higher incidence of osteoporosis and osteopenia, the association with vertebral fractures may be explained if it was hs-CRP and not BMD that was associated with the fractures. In a state of high inflammation, the mobilization of iron to the bone marrow is blocked, resulting in a condition of functional iron deficiency [22,23]. It is said that cancer patients with various tumor types exhibit a typical acute phase protein response, with increased CRP and decreased albumin, leading to high inflammation and malnutrition [24]. High inflammation, malnutrition, and iron deficiency can lead to decreased production of erythropoietin (EPO) in the kidneys and reduced responsiveness of the bone marrow to EPO, potentially causing issues in bone marrow hematopoiesis and affecting bone quality. In our previous study measuring bone marrow fat accumulation using MRI, it was suggested that BMI, as an indicator of obesity, is correlated with a transition from fatty marrow to red marrow with hematopoietic activity [25]. Jinkoo Kim et al. has demonstrated that EPO activates JAK/STAT signaling in hematopoietic stem cells (HSCs), inducing the production of bone morphogenetic protein 2 (BMP2) and promoting bone formation [26]. Additionally, EPO directly activates mesenchymal cells to form osteoblasts. The production of and responsiveness to EPO, which are essential for maintaining bone homeostasis, may also be implicated in vertebral fractures in patients with advanced prostate cancer characterized by a highly inflammatory condition.

Generally, vertebral fractures in the elderly are most common in the thoracolumbar transition (Th12–L1) and lumbar spine (L1–L5) regions [27,28,29]. While women tend to have more fractures in the thoracic spine (Th4–Th12), men are more likely to experience fractures in the lumbar spine (L1–L5) [30,31]. These differences are thought to be due to variations in bone density, lifestyle, and physical activity. In this study, PVFs evaluated using the SQ grade tended to be more common at vertebral levels Th11–L5 despite differences in grade (Figure 1). This distribution is almost consistent with the general distribution data of PVFs in elderly men. One reason existing vertebral fractures impact prognosis is that they disrupt bone alignment, leading to spinal curvature. This forward-leaning posture places additional stress on organs like the stomach, resulting in gastrointestinal and respiratory symptoms. These complications are known to reduce physical activity levels. Second, the presence of vertebral fractures may indicate the patient’s poor nutritional status. Xin-Yue Fang et al. demonstrated that mildly and moderately to severely malnourished patients were at higher risk of vertebral fractures compared to normotensive patients. Kaplan–Meier analysis showed significantly lower vertebral fracture-free survival with poor nutritional status (*p* < 0.05) [32]. There remains a lack of evidence exploring the association between PVF assessment using the SQ method and prognosis. However, it has already been used to evaluate vertebral fractures in postmenopausal women and has proven useful [33,34]. An important point is that, as Nejla El Amri suggests in her conclusion, vertebral fractures are very common in menopausal women [34]. Therefore, incorporating the SQ method into clinical and bone density measurement tools may enable more effective identification of postmenopausal fractures. The simplicity of testing is a critical factor for physicians to incorporate into their routine clinical practice, and the burden on the patient cannot be ignored. The SQ method, which is simpler than the conventional Quantitative Measurement (QM) method, does not interfere with routine clinical practice and does not impose a burden on patients. The most important thing is to identify avoidable risks as early as possible in daily clinical practice.

There are some limitations to this study that should be acknowledged. First, while the SQ method is a widely used diagnostic tool, it cannot completely eliminate the influence of the evaluator’s subjective perspective. Second, this study did not include other factors associated with osteoporosis, such as smoking, alcohol consumption, obesity, and lack of exercise. There is a report suggesting that the presence of diffuse idiopathic skeletal hyperostosis may not be negligible when assessing the risk of vertebral fracture in a prostate cancer patient before treatment [35]. Third, due to the small sample size, there is a possibility of statistical bias. Additionally, the data were obtained only from Japanese patients, and we believe that verification regarding racial differences is needed in the future.

Despite some limitations, this study provides evidence from a Japanese cohort supporting the clinical usefulness of the simpler SQ method over the QM method for evaluating vertebral fractures before starting ADT.

## 5. Conclusions

There is very limited information on the prognosis of nmPC patients with PVFs after ADT. In this study, we investigated the impact of PVF presence or absence, assessed by CT before initiating ADT, and the severity of the fracture, evaluated using the SQ method, on the survival rate of Japanese men with nmPC. During the entire observation period, 30 patients died from all causes. Multivariate Cox regression analysis identified multiple PVFs and high-grade PVF, as determined by the SQ method, as significant predictors of overall survival (OS). This analysis was adjusted for age (Model 1), and for age, Gleason score, and clinical T stage (Model 2). These findings indicate that multiple PVFs and high-grade PVFs identified before the initiation of ADT were associated with higher OS in nmPC patients treated with ADT. We believe this study supports the clinical utility of vertebral fracture assessment prior to the initiation of ADT, as recommended by current guidelines on CTIBL management.

## Figures and Tables

**Table 1:** Clinical characteristics of men with nonmetastatic prostate cancer.

Variables	Entire Cohort (*n* = 275)
Age at prostate biopsy, years	73 (55–89)
BMI, kg/m^2^	23.3 (14.3–35.7)
initial PSA, ng/mL	12.4 (4.37–716.5)
Clinical T category, *n* (%)	
T1c	41 (14.9)
T2a	64 (23.3)
T2b	68 (24.7)
T2c	64 (23.3)
T3a	22 (8.0)
T3b	7 (2.5)
T4	9 (3.3)
Clinical N category, *n* (%)	
N0	251 (91.3)
N1	24 (8.7)
Biopsy Gleason score, *n* (%)	
6	35 (12.7)
7	168 (61.1)
8	44 (16.0)
9	28 (10.2)
PVF, *n* (%)	54 (19.6)
Severity of PVFs	
Number of PVFs	
0	221 (80.4)
1	31 (11.3)
≥2	23 (8.3)
SQ grade of PVFs	
SQ = 0	221 (80.4)
SQ = 1	40 (14.5)
SQ ≥ 2	14 (5.1)
Primary treatment, *n* (%)	
ADT	115 (41.8)
ADT/radical prostatectomy	38 (13.8)
ADT/radiation therapy	122 (44.4)
Follow-up, months	55 (1–150)
Outcomes, *n* (%)	
Alive	245
Death due to prostate cancer	6
Death due to all causes	30

Data are median (range), unless otherwise indicated. Clinical staging according to American Joint Committee on Cancer TNM Staging. ADT, androgen deprivation therapy; BMI, body mass index; PSA, prostate-specific antigen; PVF, prevalent vertebral fracture; %, percentage of variables; SQ, Semiquantitative.

**Table 2:** Univariate analysis for predicting overall survival in men with nonmetastatic prostate cancer.

Variables	HR	95% CI	*p* Value
Age	1.1	1.04–1.17	0.0018
BMI	0.99	0.89–1.09	0.8341
Initial PSA	1	0.99–1.00	0.0981
PVF	4.07	1.96–8.37	0.0003
Number of PVFs			0.0007
0	1	reference	
1	3.04	1.16–7.23	
≥2	5.49	2.21–12.7	
SQ grade of PVFs			0.0006
SQ = 0	1	reference	
SQ = 1	3.34	1.45–7.37	
SQ ≥ 2	6.55	2.12–17.0	
Gleason score			0.0225
6	1	reference	
7	1.97	0.55–12.5	
8 or 9	4.8	1.31–30.8	
Clinical T stage			0.0459
T1c	1	reference	
T2a–T2c	3.02	0.88–18.9	
T3a–T4	5.66	1.41–37.7	
Clinical N stage			0.0897
N0	1	reference	
N1	2.53	0.85–6.14	

Data are median (range), unless otherwise indicated. Clinical staging according to American Joint Committee on Cancer TNM Staging. BMI, body mass index; PSA, prostate-specific antigen; PVF, prevalent vertebral fracture. SQ, Semiquantitative.

**Table 3:** Multivariate analysis for predicting overall survival in men with nonmetastatic prostate cancer.

Variables	HR	95% CI	*p* Value
Number of PVFs			
0	1	reference	
1			
Model 1	2.75	1.04–6.57	0.0414
Model 2	2.37	0.89–5.75	0.0826
≥2			
Model 1	3.81	1.45–9.37	0.008
Model 2	4.09	1.54–10.2	0.0057
SQ grade of PVFs			
SQ = 0	1	reference	
SQ = 1			
Model 1	2.78	1.19–6.23	0.0194
Model 2	2.5	1.07–5.62	0.0355
SQ ≥ 2			
Model 1	4.74	1.48–12.9	0.0115
Model 2	5.27	1.64–14.4	0.0074

PVF, prevalent vertebral fracture; SQ, Semiquantitative. Model 1; adjusted for age, Model 2; adjusted for age, Gleason score, and clinical T stage.

## Data Availability

The data presented in this study are available in this article.

## References

[1] Shao Y., Moore D.F., Shih W., Lin Y., Jang T.L., Lu-Yao G.L. (2013). Fracture after Androgen Deprivation Therapy among Men with a High Baseline Risk of Skeletal Complications. BJU Int..

[2] Watanabe D., Kimura T., Yamashita A., Minowa T., Miura K., Mizushima A. (2020). The Influence of Androgen Deprivation Therapy on Hip Geometric Properties and Bone Mineral Density in Japanese Men with Prostate Cancer and Its Relationship with the Visceral Fat Accumulation. Aging Male.

[3] Oefelein M.G., Ricchiuti V., Conrad W., Resnick M.I. (2002). Skeletal Fractures Negatively Correlate With Overall Survival in Men With Prostate Cancer. J. Urol..

[4] Burger H., Daele P.L.A.V., Grashuis K., Hofman A., Grobbee D.E., Schütte H.E., Birkenhäger J.C., Pols H.A.P. (1997). Vertebral Deformities and Functional Impairment in Men and Women. J. Bone Miner. Res..

[5] Gold D.T., Smith S.D., Bales C.W., Lyles K.W., Westlund R.E., Drezner M.K. (1991). Osteoporosis in Late Life: Does Health Locus of Control Affect Psychosocial Adaptation?. J. Am. Geriatr. Soc..

[6] Huang C., Ross P.D., Wasnich R.D. (1996). Vertebral Fracture and Other Predictors of Physical Impairment and Health Care Utilization. Arch. Intern. Med..

[7] Lyles K.W., Gold D.T., Shipp K.M., Pieper C.F., Martinez S., Mulhausen P.L. (1993). Association of Osteoporotic Vertebral Compression Fractures with Impaired Functional Status. Am. J. Med..

[8] Nevitt M.C., Ettinger B., Black D.M., Stone K., Jamal S.A., Ensrud K., Segal M., Genant H.K., Cummings S.R. (1998). The Association of Radiographically Detected Vertebral Fractures with Back Pain and Function: A Prospective Study. Ann. Intern. Med..

[9] Ross P.D., Ettinger B., Davis J.W., Melton L.J., Wasnich R.D. (1991). Evaluation of Adverse Health Outcomes Associated with Vertebral Fractures. Osteoporos. Int..

[10] Crans G.G., Silverman S.L., Genant H.K., Glass E.V., Krege J.H. (2004). Association of Severe Vertebral Fractures with Reduced Quality of Life: Reduction in the Incidence of Severe Vertebral Fractures by Teriparatide. Arthritis Rheumatol..

[11] Wang Y., Jacobs E.J., Gapstur S.M., Maliniak M.L., Gansler T., McCullough M.L., Stevens V.L., Patel A.V. (2017). Recreational Physical Activity in Relation to Prostate Cancer–Specific Mortality Among Men with Nonmetastatic Prostate Cancer. Eur. Urol..

[12] Wu C.Y., Li J., Jergas M., Genant H.K. (1995). Comparison of Semiquantitative and Quantitative Techniques for the Assessment of Prevalent and Incident Vertebral Fractures. Osteoporos. Int..

[13] Fukumoto S., Soen S., Taguchi T., Ishikawa T., Matsushima H., Terauchi M., Horie S., Yoneda T., Sugimoto T., Matsumoto T. (2020). Management Manual for Cancer Treatment-Induced Bone Loss (CTIBL): Position Statement of the JSBMR. J. Bone Miner. Metab..

[14] Huang S., Wu L., Lin S., Cai S., Zhou J. (2024). Analysis of Factors Related to Osteoporotic Vertebral Fracture in Prostate Cancer Patients. Discov. Oncol..

[15] van Oostwaard M.M., van den Bergh J.P., van de Wouw Y., Janssen-Heijnen M., de Jong M., Wyers C.E. (2023). High Prevalence of Vertebral Fractures at Initiation of Androgen Deprivation Therapy for Prostate Cancer. J. Bone Oncol..

[16] Yonou H., Aoyagi Y., Kanomata N., Kamijo T., Oda T., Yokose T., Hasebe T., Nagai K., Hatano T., Ogawa Y. (2001). Prostate-Specific Antigen Induces Osteoplastic Changes by an Autonomous Mechanism. Biochem. Biophys. Res. Commun..

[17] Kim J.-M., Lin C., Stavre Z., Greenblatt M.B., Shim J.-H. (2020). Osteoblast-Osteoclast Communication and Bone Homeostasis. Cells.

[18] Zhang X., Jiang P., Wang C. (2023). The Role of Prostate-Specific Antigen in the Osteoblastic Bone Metastasis of Prostate Cancer: A Literature Review. Front. Oncol..

[19] Ehresman J., Schilling A., Yang X., Pennington Z., Ahmed A.K., Cottrill E., Lubelski D., Khan M., Moseley K.F., Sciubba D.M. (2021). Vertebral Bone Quality Score Predicts Fragility Fractures Independently of Bone Mineral Density. Spine J..

[20] Hussain S.A., Weston R., Stephenson R.N., George E., Parr N.J. (2003). Immediate Dual Energy X-ray Absorptiometry Reveals a High Incidence of Osteoporosis in Patients with Advanced Prostate Cancer before Hormonal Manipulation. BJU Int..

[21] Eriksson A.L., Movérare-Skrtic S., Ljunggren Ö., Karlsson M., Mellström D., Ohlsson C. (2014). High-Sensitivity CRP Is an Independent Risk Factor for All Fractures and Vertebral Fractures in Elderly Men: The MrOS Sweden Study. J. Bone Miner. Res..

[22] Thomas D.W., Hinchliffe R.F., Briggs C., Macdougall I.C., Littlewood T., Cavill I. (2013). Guideline for the Laboratory Diagnosis of Functional Iron Deficiency. Br. J. Haematol..

[23] Haase V.H. (2017). HIF-prolyl Hydroxylases as Therapeutic Targets in Erythropoiesis and Iron Metabolism. Hemodial. Int..

[24] McMillan D.C. (2008). An Inflammation-Based Prognostic Score and Its Role in the Nutrition-Based Management of Patients with Cancer. Proc. Nutr. Soc..

[25] Goto K., Watanabe D., Kawae N., Nakamura T., Yanagida K., Yoshida T., Kajihara H., Mizushima A. (2024). Relationship between Femoral Proximal Bone Quality Assessment by MRI IDEAL-IQ Sequence and Body Mass Index in Elderly Men. Tomography.

[26] Kim J., Jung Y., Sun H., Joseph J., Mishra A., Shiozawa Y., Wang J., Krebsbach P.H., Taichman R.S. (2012). Erythropoietin Mediated Bone Formation Is Regulated by MTOR Signaling. J. Cell. Biochem..

[27] Akeda K., Nakase K., Yamada J., Takegami N., Fujiwara T., Sudo A. (2024). Progression of Vertebral Deformity of Prevalent Vertebral Fractures in the Elderly: A Population-Based Study. BMC Musculoskelet. Disord..

[28] Zhang Y.-K., Wang J.-X., Ge Y.-Z., Wang Z.-B., Zhang Z.-G., Zhang Z.-W., Chang F. (2025). The Global Burden of Vertebral Fractures Caused by Falls among Individuals Aged 55 and Older, 1990 to 2021. PLoS ONE.

[29] Freitas S.S., Barrett-Connor E., Ensrud K.E., Fink H.A., Bauer D.C., Cawthon P.M., Lambert L.C., Orwoll E.S., Osteoporotic Fractures in Men (MrOS) Research Group (2008). Rate and Circumstances of Clinical Vertebral Fractures in Older Men. Osteoporos. Int..

[30] Cawthon P.M. (2011). Gender Differences in Osteoporosis and Fractures. Clin. Orthop. Relat. Res..

[31] Duan Y., Turner C.H., Kim B., Seeman E. (2009). Sexual Dimorphism in Vertebral Fragility Is More the Result of Gender Differences in Age-Related Bone Gain Than Bone Loss. J. Bone Miner. Res..

[32] Fang X.-Y., Xu H.-W., Chen H., Zhang S.-B., Yi Y.-Y., Ge X.-Y., Wang S.-J. (2023). Association Between Poor Nutritional Status and Increased Risk for Subsequent Vertebral Fracture in Elderly People with Percutaneous Vertebroplasty. Clin. Interv. Aging.

[33] Cipriani C., Minisola S., Bilezikian J.P., Diacinti D., Colangelo L., Piazzolla V., Angelozzi M., Nieddu L., Pepe J., Diacinti D. (2021). Vertebral Fracture Assessment in Postmenopausal Women With Postsurgical Hypoparathyroidism. J. Clin. Endocrinol. Metab..

[34] Amri N.E., Daldoul C., Lataoui S., Baccouche K., Belghali S., Zeglaoui H., Bouajina E. (2021). Asymptomatic Vertebral Fracture in Tunisian Post-Menopausal Women at Risk: Prevalence and Risk Factors. Arch. Osteoporos..

[35] Watanabe D., Takano H., Kimura T., Yamashita A., Minowa T., Mizushima A. (2020). The Relationship of Diffuse Idiopathic Skeletal Hyperostosis, Visceral Fat Accumulation, and Other Age-Related Diseases with the Prevalent Vertebral Fractures in Elderly Men with Castration-Naïve Prostate Cancer. Aging Male.

